# Supplementary material for: Embryonic Stem Cell-Derived Neurons as a Model System for Epigenome Maturation during Development
Source: Genes (Basel). 2023 Apr 22;14(5):957. doi: 10.3390/genes14050957 (PMC10218269; doi:10.3390/genes14050957)
Supplement: Supplementary file 1 [file genes-14-00957-s001.zip › genes-2270578-supplementary.pdf]

Supplementary Materials

Embryonic stem cell-derived neurons as a model system for epigenome maturation during development

Sally Martin[#], Daniel Poppe[#], Nelly Olova, Conor O'Leary, Elena Ivanova, Jahnvi Pflueger, Jennifer Dechka, Rebecca K. Simmons, Helen M. Cooper, Wolf Reik, Ryan Lister[*] and Ernst J. Wolvetang[*]

Figure S1: Characterisation of mESC-derived neurons

Figure S2: Specificity of the anti-mCA antibody determined by ICC

Figure S3: Identification and isolation of neuronal nuclei in human PSC-derived neurons and mouse ESC-derived neurons

Figure S4: Correlation of gene length and DNA methylation between *in vivo* and *in vitro* neurons

Figure S5: mCG and mCH within genes in ESC-derived and iN cells compared to *in vivo* neurons

Figure S6: mCH and mCG within genes sorted for differences in the other context between neuronal samples

Figure S7: Top 50 terms for genes differentially methylated in CG context between neuronal populations

Figure S8: Top 50 terms for genes differentially methylated in CH context between neuronal populations

Figure S9: Enrichment for genes with similar methylation patterns for *in vitro* neurons and *in vivo* neurons

Figure S10: ChIP-seq data for K27me3 and K36me3 compared to methylation and RNA expression along gene bodies

Figure S11: GO terms for genes linked to cortical enhancers with high CG methylation *in vitro*

Table S1: Antibodies

Table S2: RT-qPCR primers

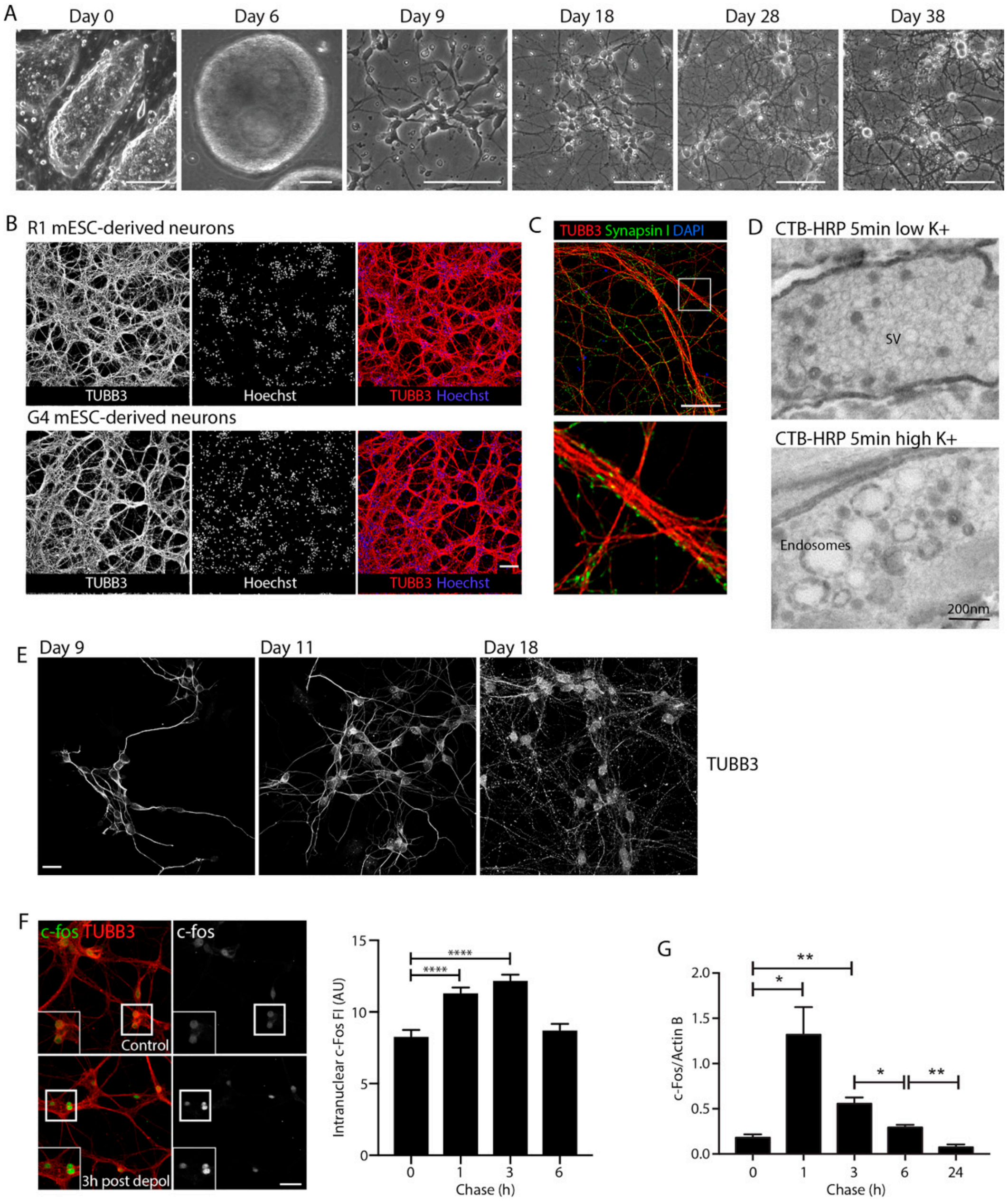

Figure S1. Characterisation of mESC-derived neurons. (**a**) Phase contrast images of mESCs growing on feeder MEFs, cell aggregates (day 6), neural progenitors (day 9), and neurons (day 18-38). Scale bar =100 µm, except for cell aggregates = 200 µm. (**b**) Neurons derived from either G4 or R1 mESCs labelled for the pan-neuronal marker beta3-tubulin (TUBB3). Both cell lines generated complex neurite networks within 38 days. Scale bar = 100 µm. (**c**) Neurons derived from G4 mESCs labelled for TUBB3 and pre-synaptic protein synapsin 1. Punctate labelling for Syn1 along neurites demonstrates the presence of nascent synapses. Scale bar = 50 µm. (**d**) Depolarisation response of synapses was shown by transmission electron microscopy. R1 mESC-derived neurons were incubated for 5 min with CTB-HRP in either low or high K+ buffer. Low levels of tracer endocytosis into synaptic vesicles in low K+ was superseded by high levels of bulk endocytosis in depolarised cells, suggesting a strong rapid burst of neuroexocytosis and compensatory endocytosis (51). (**e**) G4 mESC-derived neurons labelled for TUBB3 at different times during differentiation. Scale bar = 20 µm. (**f**) G4 mESC-derived neurons were depolarised for 5 min with high K+, then chased in growth medium for 1-6 h. Cells were labelled for c-Fos and TUBB3. The intranuclear fluorescence intensity FI of c-Fos was determined during the chase. Results = mean ± SEM, for one representative differentiation. Similar labelling profiles were observed in two separate differentiations. Depolarisation resulted in a transient increase in intranuclear c-Fos labelling in the post-depolarisation period. **** $p<0.0001$, Student's t-test. Scale bar = 50 µm. (**g**) c-Fos mRNA abundance was determined by RT-qPCR in control G4 mESC-derived neurons and during a 24 h chase following a 5 min transient depolarisation by high K+. Results shown are mean ± SEM relative to beta-actin levels. N=3 independent experiments. * $p<0.05$, ** $p<0.01$, Student's t-test.

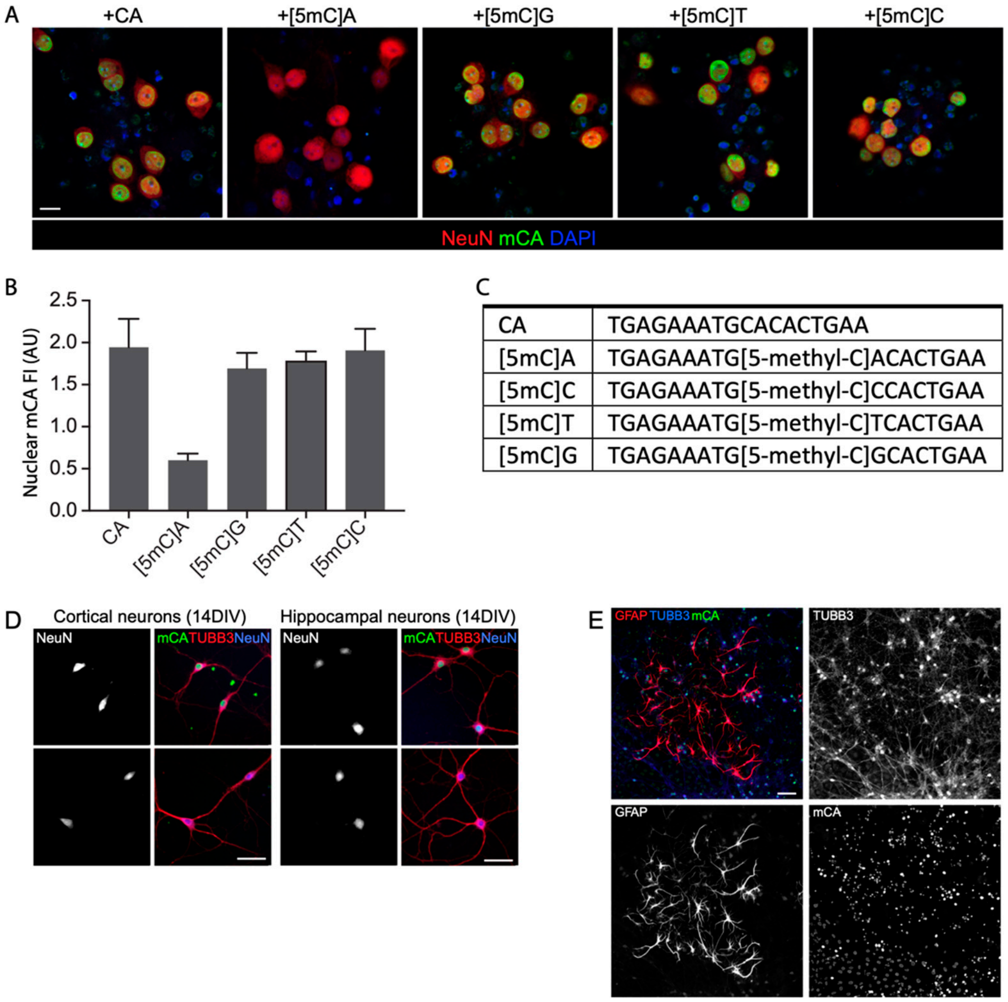

Figure S2. Specificity of the anti-mCA antibody determined by ICC. (**a**) mESC-derived neurons were fixed and immunolabelled for NeuN and mCA ± 2.5 μM competitive methylated oligonucleotides: [5mC]A, [5mC]G, [5mC]T and [5mC]C, or the non-methylated CA oligonucleotide. Scale bar = 10μm. (**b**) The intranuclear fluorescence intensity (FI, arbitrary units) of the nuclear mCA labelling in NeuN-masked nuclei was determined. (**c**) Sequence of the methylated oligonucleotides. (**d**) Related to Figure 1C: 14DIV cortical or hippocampal neurons immunolabelled for NeuN, TUBB3 and mCA with blue channel (NeuN) additionally shown in greyscale. Scale bar = 50 μm. (**e**) Related to Figure 1F: Later stage neural differentiations (Day30-38) immunolabelled for astrocytes (GFAP+), neurons (TUBB3+) and mCA, showing all three channels in greyscale. Scale bar = 50 μm.

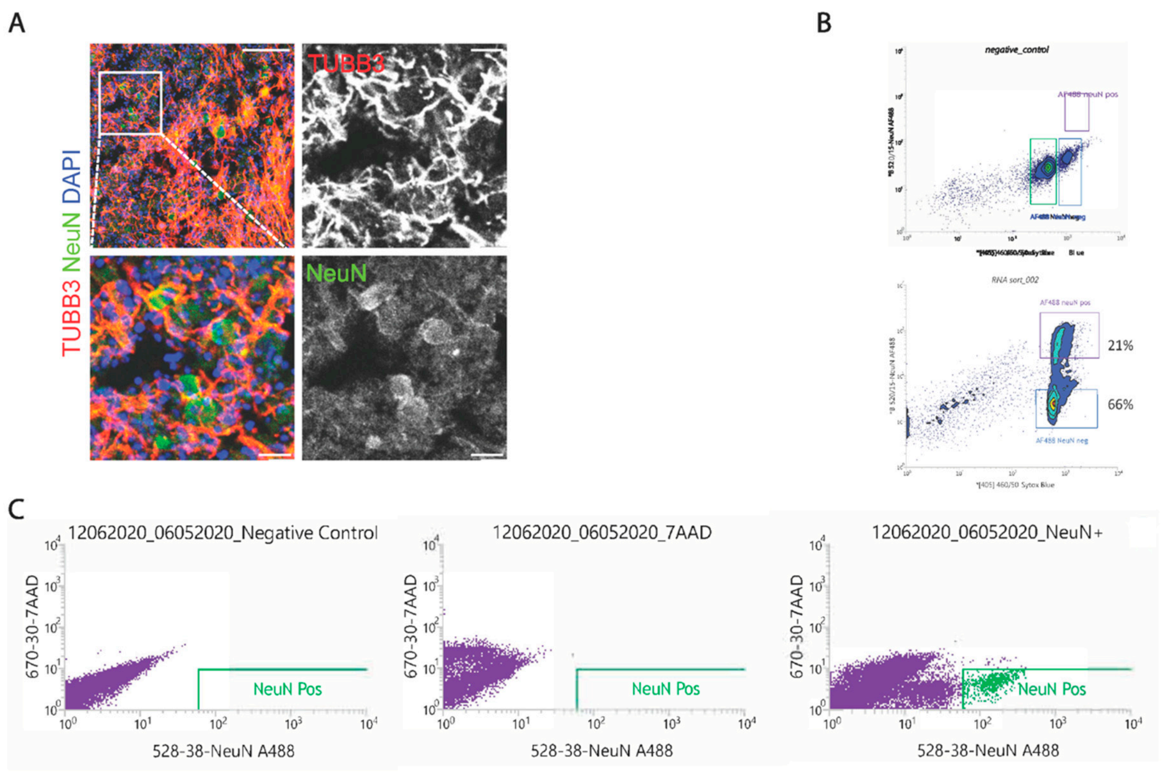

Figure S3. Identification and isolation of neuronal nuclei in human PSC-derived neurons and mouse ESC-derived neurons. (**a**) Immunohistochemistry and (**b**) FANS analyses confirmed the presence of NeuN +ve nuclei in human PSC-derived cortical organoids cultured for 3 months. Scale bar: 100 µm top left, 20 µm all others. (**c**) NeuN +ve nuclei sorted from mESC-derived neuronal cultures. Panels show negative control (no antibody, left), 7-AAD (nuclei stain, middle) only, and co-labelling for 7-AAD and NeuN (right).

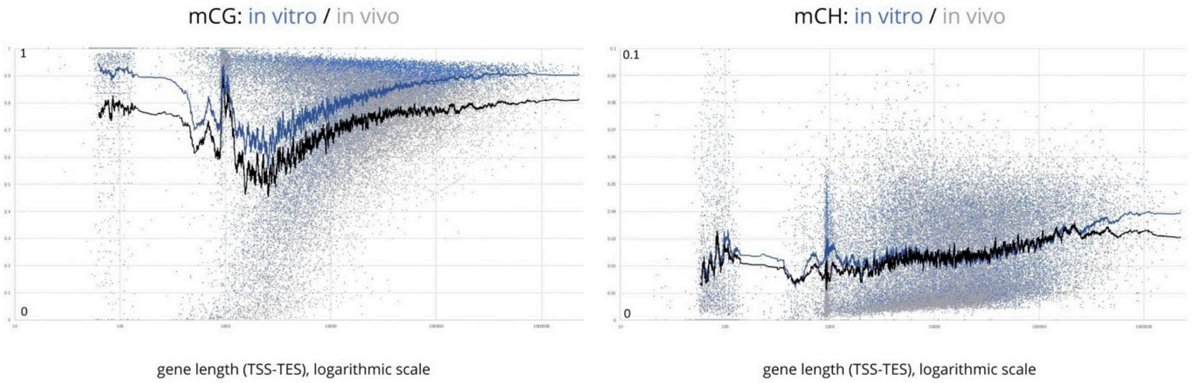

Figure S4. Correlation of gene length and DNA methylation between *in vivo* and *in vitro* neurons. Dot plots showing gene length from transcription start site (TSS) to transcription end site (TES) in logarithmic scale on x-axis and average methylation of gene bodies for CG and CH context on y-axis. *In vitro* neurons in blue and *in vivo* neurons in black. Line shows sliding average over 20 genes.

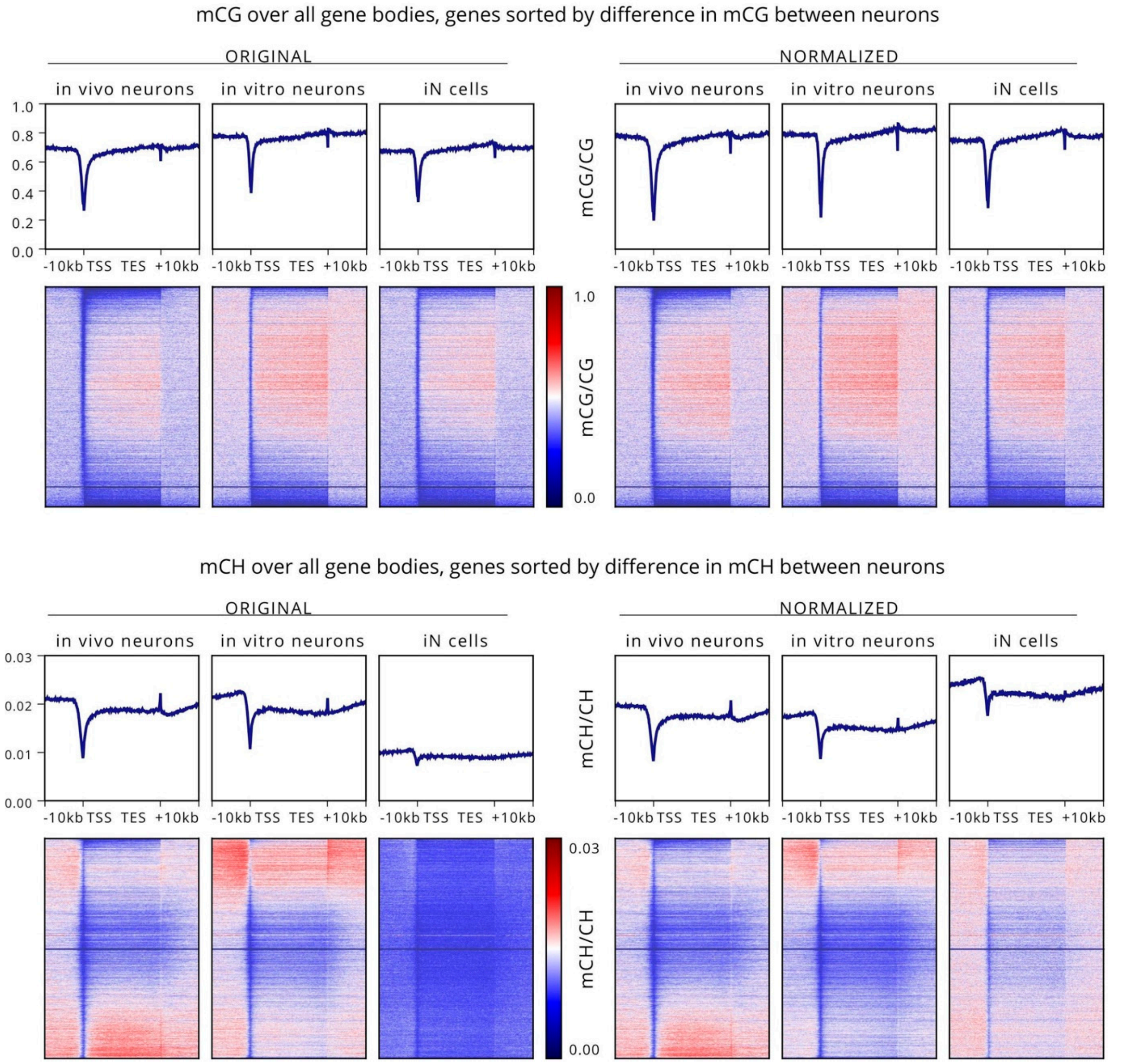

Figure S5. mCG and mCH within genes in ESC-derived and iN cells compared to *in vivo* neurons. Genes in the same order based on methylation difference as in Figure 5A and Figure 6A but showing methylation for gene bodies and flanking 10 kb for 7-week adult mouse prefrontal cortex neurons (*in vivo* neurons), mESC-derived neurons (*in vitro* neurons), and trans-differentiated induced neurons (iN) cells as reported by Luo *et al*. [55]. Left side = original data, right side = normalized to average global CG or CH methylation levels.

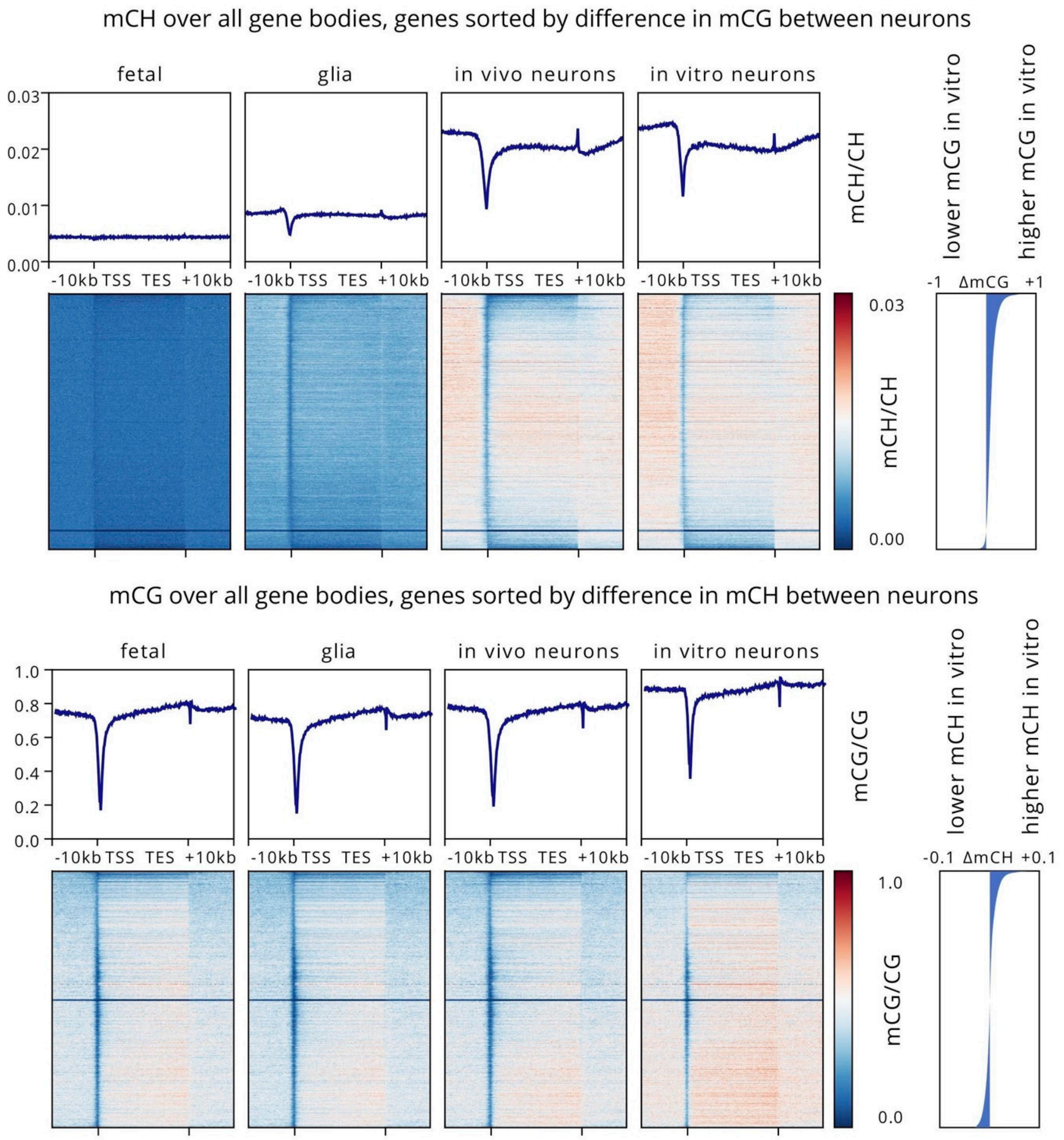

Figure S6. mCH and mCG within genes sorted for differences in the other context between neuronal samples. Genes in the same order based on methylation difference as in Figure 5A and Figure 6A but showing methylation for the other context in gene bodies and flanking 10 kb for fetal mouse frontal cortex (fetal), NeuN-negative cells from 7-week adult mouse prefrontal cortex (glia), 7-week adult mouse prefrontal cortex neurons (*in vivo* neurons), and d38 mESC-derived neurons (*in vitro* neurons). Difference in methylation between both neuronal samples used for gene order is shown on the right.

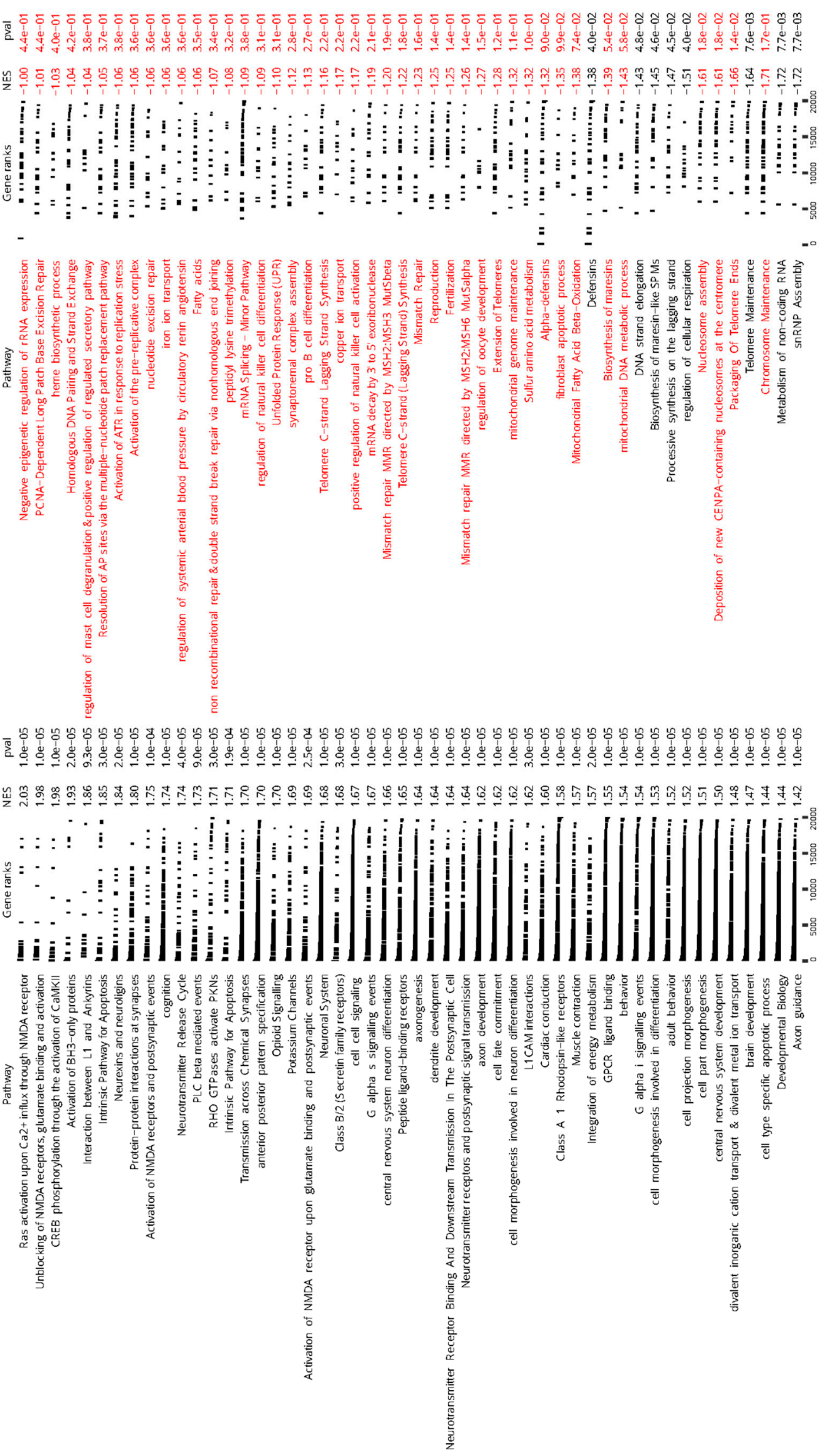

Figure S7. Top 50 terms for genes differentially methylated in CG context between neuronal populations. Pathways were ranked by enrichment score (NES), where positive NES indicates pathways enriched in genes hypermethylated for CG and negative NES indicates pathways enriched in hypomethylated genes, *in vitro* neurons. Gene rank plots show position of genes being part of a pathway set within the ordering of all genes based on methylation difference in mCG. Pathways with p values larger than 0.05 are shown in red.

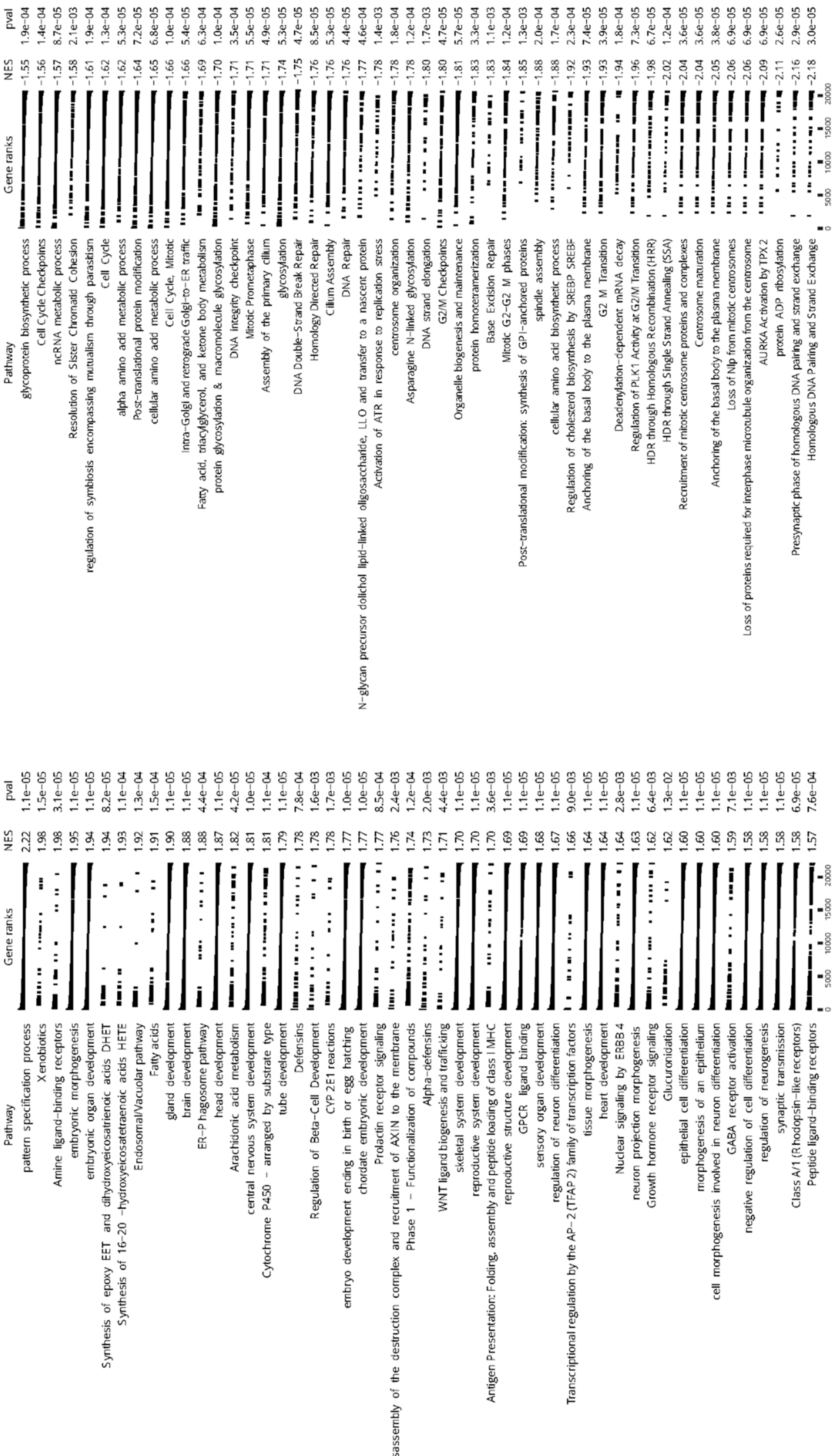

Figure S8. Top 50 terms for genes differentially methylated in CH context between neuronal populations. Pathways were ranked by enrichment score (NES), whereas positive NES indicates pathways enriched in genes hypermethylated for CH in *in vitro* neurons, while negative NES indicates pathways enriched in hypomethylated genes. Gene rank plots show the position of genes being part of a pathway set within the ordering of all genes based on methylation difference in mCH.

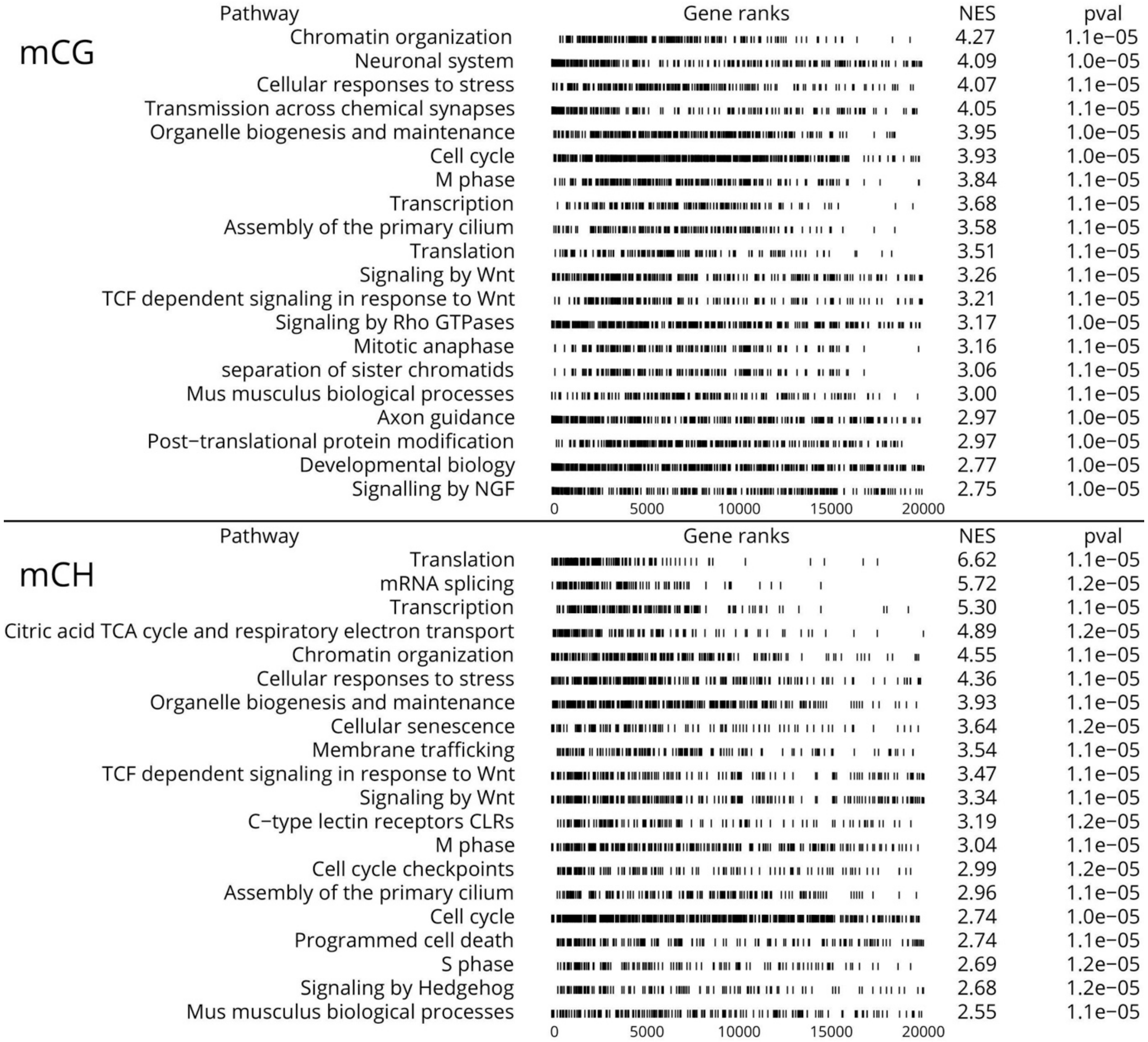

Figure S9. Enrichment for genes with similar methylation patterns for *in vitro* neurons and *in vivo* neurons. Pathways were ranked by enrichment score (NES), based on similarity between *in vitro* and *in vivo* neurons and dissimilarity to glia and fetal brain in mCG and mCH context respectively. Gene rank plots show the position of genes being part of a pathway set within the ranking of all genes.

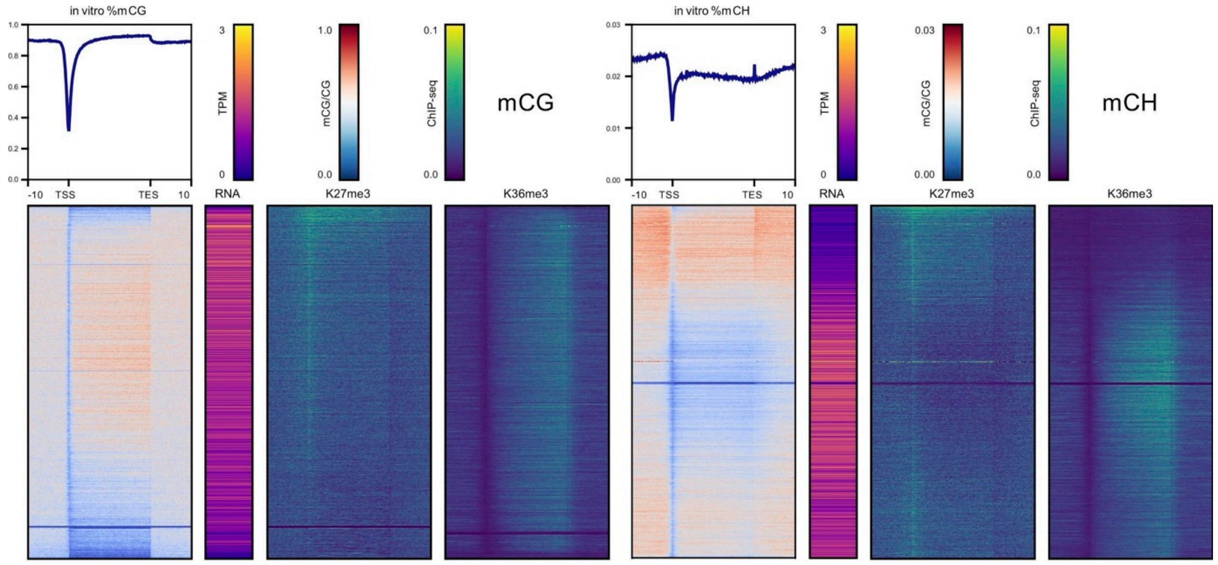

Figure S10. ChIP-seq data for K27me3 and K36me3 compared to methylation and RNA expression along gene bodies. mCG data on the left, mCH data on the right. Shown are TPM values from RNA-seq data as well as ChIP-seq data from Lienert *et al*. [63] for K27me3 and Tippmann *et al*. [64] for K36me3, for all gene bodies in the same order as shown in Figure 5A and Figure 6A.

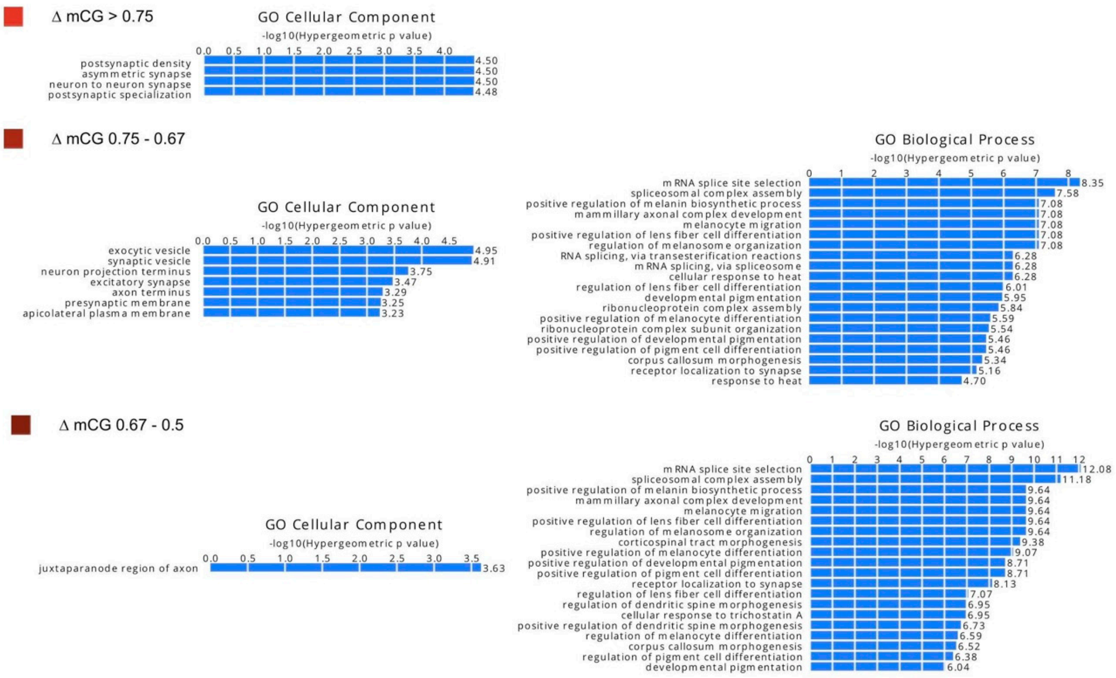

Figure S11. GO terms for genes linked to cortical enhancers with high CG methylation *in vitro*. mCG data on the left, mCH data on the right. Same methylation difference brackets were used as in Figure 7F. Enrichment was performed with GREAT using all genes linked to any cortical enhancer as background.

Table S1. Antibodies

Target	Host	Clone	Supplier	Catalog #
TUBB3 (Beta III Tubulin)	Rabbit		Sigma-Aldrich	T2200
TUBB3 (Beta III Tubulin)	Chicken		Merck-Millipore	AB9354
NeuN (ICC)	Rabbit	D4G4O	Cell Signaling Technology	24307
NeuN (IHC)	Rabbit		Abcam	ab128886
NeuN-Alexa488	Mouse	A60	Merck-Millipore	MAB377X
c-Fos	Guinea pig		Synaptic Systems	226 004
Synapsin 1	Rabbit		Merck-Millipore	AB154
Pax6	Rabbit	Poly19013	BioLegend	19013
5-mC	Rabbit	RM231	Abcam	ab214727
5-hmC	Rabbit		Active Motif	39769
mCA	Mouse	2C8H8A6	Active Motif	61783/4
mCG	Mouse	3A7	Reik Laboratory, Babraham Institute	-
GFAP	Rabbit		Dako	Z0344
Nanog	Rabbit	D2A3	Cell Signaling Technology	8822
**Secondary antibody**	**Host**	**Fluorophore**	**Supplier**	**Catalog #**
Anti-chicken	Donkey	Alexa568	Invitrogen	A78950
Anti-chicken	Donkey	Alexa647	Invitrogen	A78952
Anti-guinea pig	Goat	Alexa647	Invitrogen	A21450
Anti-mouse	Donkey	Alexa488	Invitrogen	A32766
Anti-mouse	Donkey	Alexa568	Invitrogen	A10037
Anti-rabbit	Donkey	Alexa568	Invitrogen	A10042
Anti-rabbit	Donkey	Alexa647	Invitrogen	A31673

Table S2. RT-qPCR primers

Target	Gene ID	Forward primer	Reverse primer	Ref.
beta-Actin	11461	AAGATCAAGATCATTGCTCCTCCT	CAGCTCAGTAACAGTCCGCC	-
NeuN/RbFox3	52897	ATCGTAGAGGGACGGAAAATTGA	GTTCCCAGGCTTCTTATTGGTC	-
Dnmt1	13433	AAGAATGGTGTTGTCTACCGAC	CATCCAGGTTGCTCCCCTTG	[96]
Dnmt3a	13435	GATGAGCCTGAGTATGAGGATGG	CAAGACACAATTCGGCCTGG	-
c-Fos	14281	CCTACTACCATTCCCCAGCC	CTGTCACCGTGGGGATAAAG	[97]
